# *GNS4*, a novel allele of *DWARF11*, regulates grain number and grain size in a high-yield rice variety

**DOI:** 10.1186/s12284-017-0171-4

**Published:** 2017-07-20

**Authors:** Yong Zhou, Yajun Tao, Jinyan Zhu, Jun Miao, Jun Liu, Yanhua Liu, Chuandeng Yi, Zefeng Yang, Zhiyun Gong, Guohua Liang

**Affiliations:** 1 Jiangsu Key Laboratory of Crop Genetics and Physiology/Co-Innovation Center for Modern Production Technology of Grain Crops, Key Laboratory of Plant Functional Genomics of the Ministry of Education, Yangzhou University, Yangzhou, 225009 China; 2 Institute of Food Crops, Jiangsu Academy of Agricultural Sciences, Nanjing, 210014 China

**Keywords:** Rice, *GNS4/D11*, Grain number, Grain size, Cell elongation

## Abstract

**Background:**

Rice plays an extremely important role in food safety because it feeds more than half of the world’s population. Rice grain yield depends on biomass and the harvest index. An important strategy to break through the rice grain yield ceiling is to increase the biological yield. Therefore, genes associated with organ size are important targets for rice breeding.

**Results:**

We characterized a rice mutant *gns4* (*grain number and size on chromosome 4*) with reduced organ size, fewer grains per panicle, and smaller grains compared with those of WT. Map-based cloning indicated that the *GNS4* gene, encoding a cytochrome P450 protein, is a novel allele of *DWARF11* (*D11*). A single nucleotide polymorphism (deletion) in the promoter region of *GNS4* reduced its expression level in the mutant, leading to reduced grain number and smaller grains. Morphological and cellular analyses suggested that *GNS4* positively regulates grain size by promoting cell elongation. Overexpression of *GNS4* significantly increased organ size, 1000-grain weight, and panicle size, and subsequently enhanced grain yields in both the Nipponbare and Wuyunjing7 (a high-yielding cultivar) backgrounds. These results suggest that *GNS4* is key target gene with possible applications in rice yield breeding.

**Conclusion:**

*GNS4* was identified as a positive regulator of grain number and grain size in rice. Increasing the expression level of this gene in a high-yielding rice variety enhanced grain yield. *GNS4* can be targeted in breeding programs to increase yields.

**Electronic supplementary material:**

The online version of this article (doi:10.1186/s12284-017-0171-4) contains supplementary material, which is available to authorized users.

## Background

The world’s population is estimated to grow to around 8.5 billion by 2030 (http://esa.un.org/unpd/wpp/Publications/). To feed the growing population, it is estimated that agricultural production needs to increase by 60% (Yamaguchi and Hwang 2015). Rice (*Oryza sativa* L.) is a staple food of more than 3.5 billion people, mainly in Asia (Seck et al. 2012). A significant improvement in rice yield per unit ground area would significantly reduce the global food shortage.

Rice grain yield is defined as the product of yield sink capacity and filling efficiency (Kato and Takeda 1996). To achieve new breakthroughs in yield, breeding efforts have focused on expanding the yield sink capacity, mainly by increasing the number of grains per panicle and grain size. Strategies including high fertilizer inputs and optimized cultivation methods have been used to increase grain number and enhance grain filling to maximize rice production. New varieties, especially the so-called ‘super rice’ cultivars that produce large numbers of grains per panicle with a large yield potential have been bred and cultivated. There have also been breakthroughs in elucidating the molecular mechanisms underlying rice yield traits. Using molecular genetic approaches, researchers have identified several genes that control the size of rice panicle and grain. For example, mutants of *LAX1*, *FZP*, *LOG*, *APO1*, *SP1*, *FON*, *DEP2*, *DEP3*, and *PAY1* genes were found to produce abnormal inflorescences and smaller panicles (Chu et al. 2006; Ikeda et al. 2007; Komatsu et al. 2003; Komatsu et al. 2001; Kurakawa et al. 2007; Li et al. 2010; Li et al. 2009; Qiao et al. 2011; Suzaki et al. 2004; Zhao et al. 2015). Several quantitative trait loci (QTL) controlling grain number have been identified. Among them, *Gn1a*, *IPA1*, *PROG1*, *An-1*, and *An-2* negatively regulate grain number per panicle (Ashikari et al. 2005; Gu et al. 2015; Jiao et al. 2010; Jin et al. 2008; Luo et al. 2013), while *SPIKE* and *qGP5–1* are related to increased grain number (Dong et al. 2013; Fujita et al. 2013).

Rice grain size is defined by grain length, width, length-width ratio, and grain weight, and is another important factor in determining rice yield. Generally, dwarf mutants of the genes involved in gibberellin (GA) and brassinosteroid (BR) biosynthesis and signaling, such as *D1*, *D2*, *D11*, *D18*, *D61*, *BRD1*, *BRD2*, and *DSG1*, produce smaller grains (Ashikari et al. 1999; Hong et al. 2003; Itoh et al. 2001; Mori et al. 2002; Tanabe et al. 2005; Yamamuro et al. 2000). Several QTL related to grain size have been isolated. For example, *GS3*, *GL3.1*, *GW2*, *GW5* and *GS5* control grain size (Fan et al. 2006; Li et al. 2011; Qi et al. 2012; Song et al. 2007; Weng et al. 2008), *GW8* and *GL7/GW7/SLG7* regulate grain shape (Wang et al. 2015a; Wang et al. 2012; Wang et al. 2015b; Zhou et al. 2015), and *GIF1* and *TGW6* control grain filling (Ishimaru et al. 2013; Wang et al. 2008).

In this study, we characterized a rice mutant, *gns4* (*grain number and size on chromosome 4*), which showed reduced grain number per panicle and smaller grains compared with those of wild type (WT). The *GNS4* gene, isolated via a map-based cloning approach, was found to be a novel allele of *DWARF11* (*D11*), which encodes a cytochrome P450 protein. *GNS4* regulates the expression levels of genes involved in BR synthesis and BR response. Overexpression of *GNS4* in a high-yielding cultivar background significantly enhanced grain weight and increased grain yield, suggesting that *GNS4* is key target gene with possible applications in yield breeding.

## Results

### Characters of *gns4* mutant

**Fig. 1:**
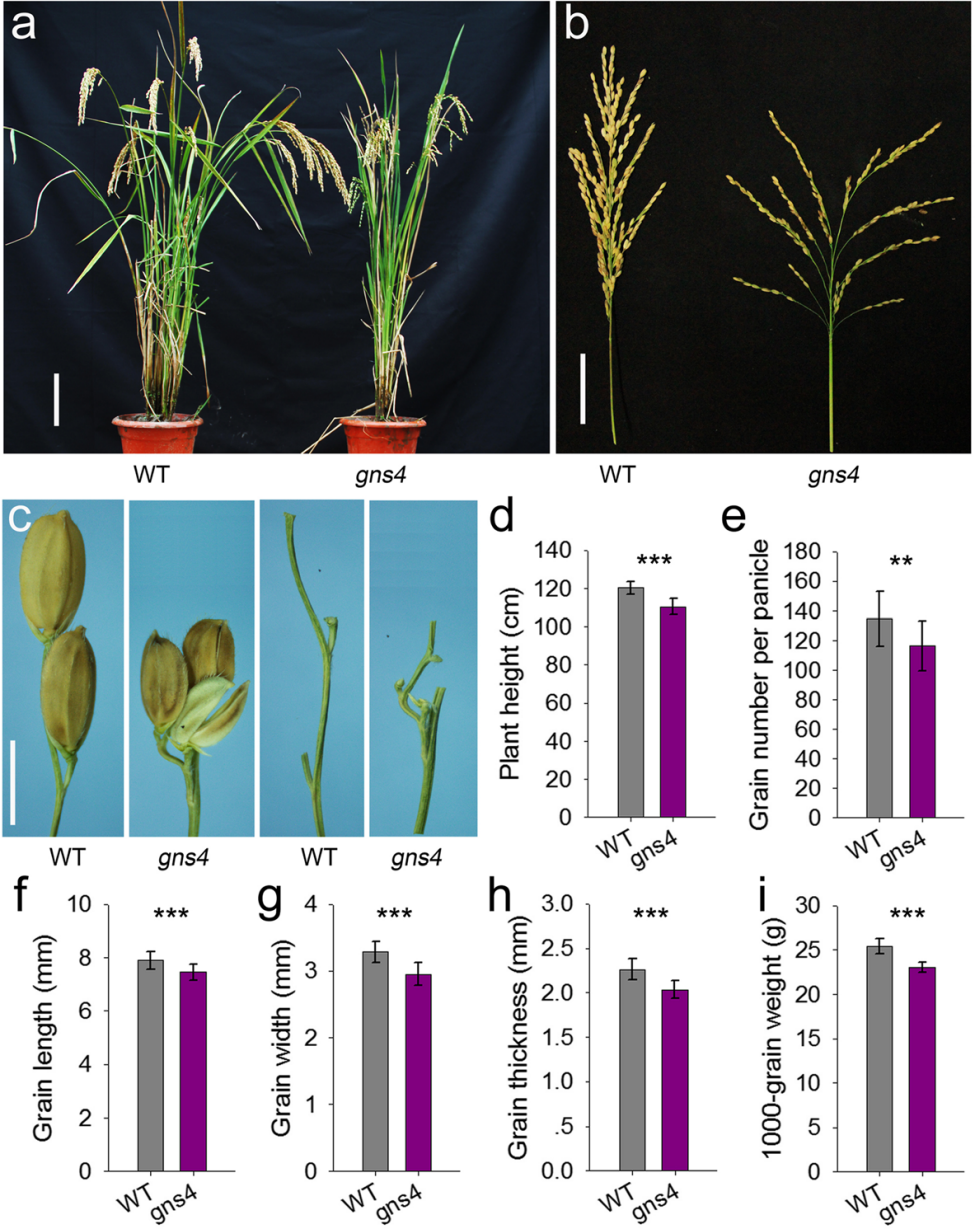
Morphological characteristics of the *gns4* mutant. **a** Gross morphologies of WT and *gns4* mutant at mature stage. Scale bar, 15 cm. **b** Panicle phenotypes of WT and *gns4* mutant. Scale bar, 5 cm. **c** Grains and pedicels of WT and *gns4* mutant. Scale bar, 5 mm. **d-i** Comparison of agronomic traits between WT and *gns4* mutant. Data are given as means ± s.e. (*n* ≥ 20). Significant at ***0.1% and **1%

To investigate the mechanism underlying panicle and grain development in rice, we conducted a genetic screen for mutants with altered panicle and grain size. The *gns4* mutant was isolated from EMS-treated *japonica* variety Zhonghua 11C (ZH11C). At maturity, *gns4* plants were shorter than WT plants (Fig. 1a, d), and produced smaller panicles and grains than those of WT (Fig. 1b, c). The average grain number per panicle of *gns4* was 86.4% of that in ZH11C (Fig. 1e). As well as the reduced grain number, the main axes of *gns4* were vestigial. The degree of spikelet clustering mainly depended on the length of the secondary branches. Some secondary branches of the *gns4* mutant were significantly shortened, which caused spikelet clustering. The grain length, grain width, and grain thickness were significantly smaller in the *gns4* mutant than in WT (Fig. 1f–h), resulting in reduced 1000-grain weight (9.4% lower than that of WT) (Fig. 1i). Together, these results indicated that *GNS4* influences panicle and grain size in rice.

### Map-based cloning of *GNS4*

To investigate the genetic basis of the mutation, we crossed *gns4* with an *indica* variety 9311 to develop F_1_ and F_2_ populations. All the F_1_ plants showed a wild type phenotype. In the F_2_ population, plants with the WT and mutant phenotypes conformed to a 3:1 segregation ratio (*X* ^2^ = 0.98 < *X* ^2^_0.05_ = 3.84), suggesting that this mutation was controlled by a single recessive gene.

**Fig. 2:**
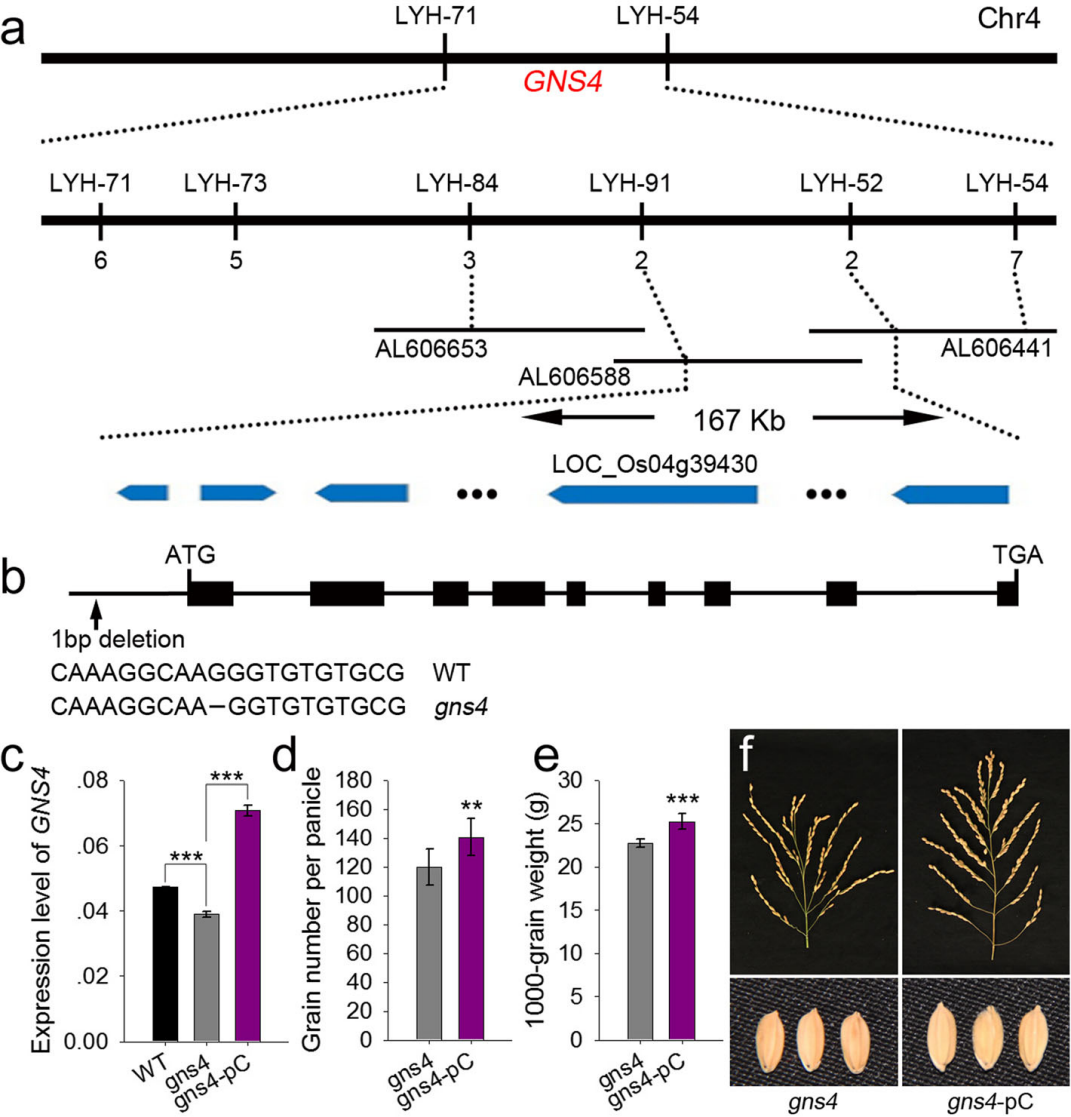
Map-based cloning of *GNS4*. **a** Fine mapping of *GNS4*. *GNS4* was primarily mapped between markers LYH-54 and LYH-71 on chromosome 4. Then, *GNS4* was fine mapped into a 167-kb segment between markers LYH-52 and LYH-91, using 856 recessive plants from the segregating population. The numbers underneath each marker indicate the numbers of recombinants between *GNS4* and the molecular markers. The candidate genes within this region were showed in blue. **b** Gene structure and the sequence variance of *GNS4* between WT and the *gns4* mutant. **c** The expression levels of *GNS4* among WT, *gns4* and the complementary transgenic plants. The expression level of the rice *Actin* gene was amplified as a control. Values are means ± s.e. of three independent experiments. **d** Comparison of grain number per panicle between *gns4* mutant and complementary transgenic plants. Data are given as means ± s.e. (*n* = 11). **e** Comparison of grain weight between *gns4* mutant and complementary transgenic plants. Data are given as means ± s.e. (*n* = 11). **f** Panicle and grain phenotypes of *gns4* and complementary transgenic plants. gns4-pC presents the transgenic *gns4* plants with pGNS4::GNS4^ZH11C^ construct. Significant at ***0.1% and **1%

We then isolated the *GNS4* gene using map-based cloning. Firstly, the *gns4* gene was limited between two molecular markers, LYH-71 and LYH-54, on chromosome 4 (Fig. 2a). The *gns4* mutation was further fine-mapped to a 167-kb interval between the markers LYH-91 and LYH-52. Within this chromosome segment, there were 24 predicted open reading frames (ORFs). We failed to develop more polymorphic markers to further narrow down the candidate region, so we sequenced and analyzed all 24 ORFs. Only one SNP in the promoter region of LOC_Os04g39430 differed between WT and the *gns4* mutant (Fig. 2b). The transcript level of LOC_Os04g39430 was significantly lower in *gns4* leaves than in WT leaves (Fig. 2c), suggesting that LOC_Os04g39430 was a good candidate for *GNS4*.

To test this prediction, we generated a plasmid expressing the LOC_Os04g39430-coding region under the control of its native promoter. We introduced this construct into the *gns4* mutant by *Agrobacterium*-mediated transformation. Twelve transgenic plants were generated, and all the positive lines showed complementation of the mutant phenotypes (Fig. 2d–f). This result confirmed that LOC_Os04g39430 was *GNS4*.

Next, a BLASTP analysis revealed that *GNS4* was allelic to *DWARF 11* (*D11*), which encodes a cytochrome P450 superfamily protein CYP724B1 (Tanabe et al. 2005). A previous study showed that *D11* plays a role in BR synthesis and may be involved in the supply of typhasterol (TY) and 6-deoxoTY to the BR synthesis network in rice. In the *gns4* mutant, a nucleotide deletion in the promoter region of *GNS4* caused reduced grain number and smaller grains. We compared the transcript levels of *GNS4* between WT and the *gns4* mutant, and observed a slightly lower level of *GNS4* transcripts in the mutant. Because the leaf phenotype of *gns4* was not as stiff as those of the *d11–1* and *d11–2* mutants, we concluded that *gns4* was likely to be a weak mutation of *D11*.

### Manipulation of *GNS4* has large effects on grain number and grain size

**Fig. 3:**
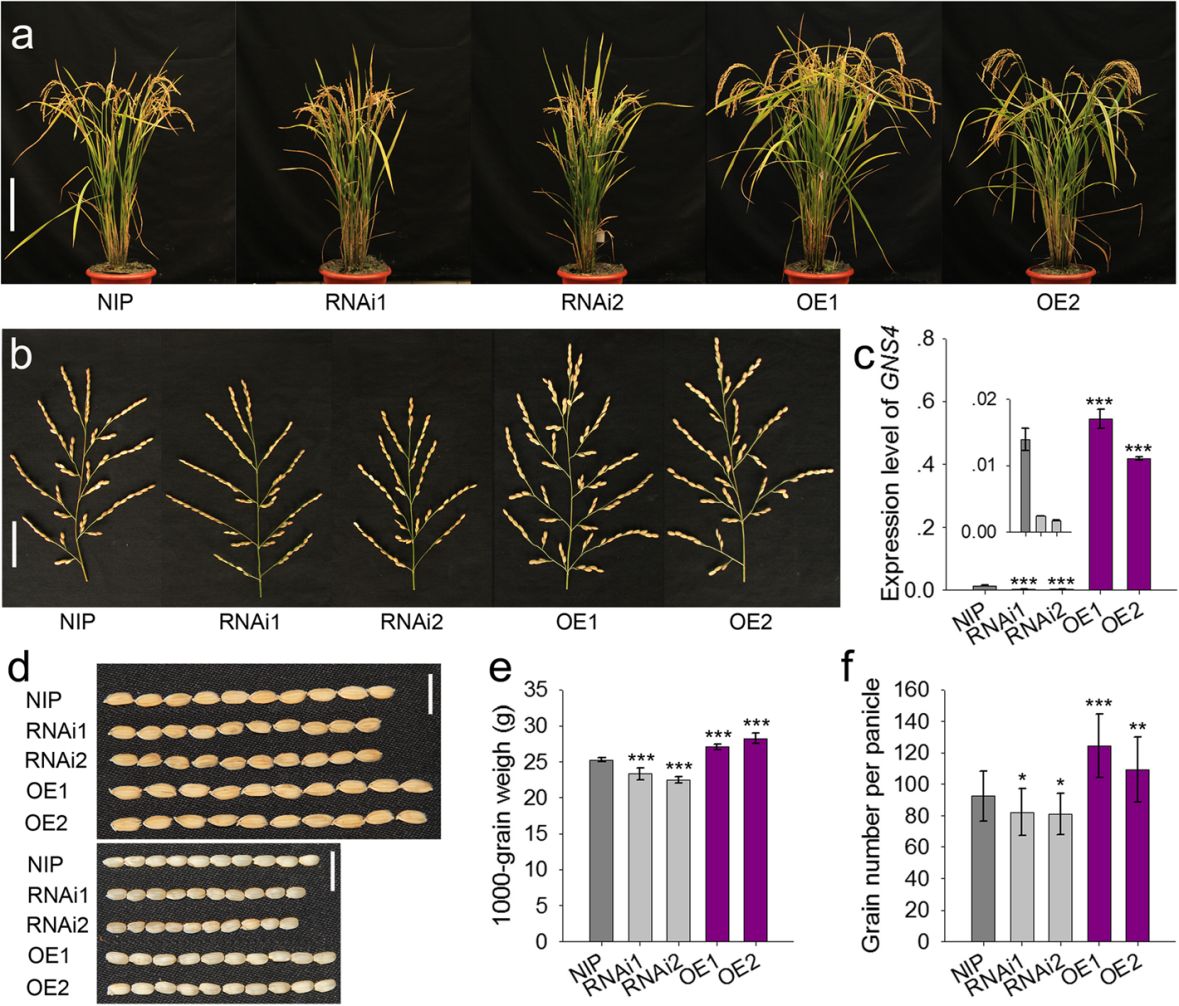
RNAi and overexpression analysis of *GNS4* gene in Nipponbare background. **a** Phenotypic features of *GNS4* RNAi and overexpression transgenic plants. Bar, 20 cm. **b** Panicle architecture of *GNS4* RNAi and overexpression transgenic plants. Bar, 5 cm. **c** *GNS4* mRNA expression level of the RNAi and overexpression transgenic plants. The expression level of the rice *Actin* gene was amplified as a control. Values are means ± s.e. of four independent experiments. **d** Grains of the transgenic plants. Bar, 1 cm. **e-f** Comparison of 1000-grain weight and grain number per plant between Nipponbare and transgenic lines. Data are given as means ± s.e. (*n* ≥ 13). Significant at ***0.1%, **1% and *5%. NIP, Nipponbare. RNAi1 and RNAi2 are two independent lines for RNAi analysis, and OE1 and OE2 are two independent lines for overexpression analysis

To further determine the roles of *GNS4* in panicle and grain development, we created transgenic plants in Nipponbare (NIP) background by expressing a *pUbi:RNAi–GNS4* construct driven by a constitutively expressed maize ubiquitin promoter. The positive lines (RNAi1 and RNAi2) with down-regulated *GNS4* expression displayed a semi-dwarf stature (Fig. 3a, c). Similar to the *gns4* mutant, the RNAi1 and RNAi2 plants had shortened plant height and panicle length (Table 1), and fewer grains per panicle (10.3% and 11.1% lower than that in WT, respectively) (Fig. 3b). Compared with Nipponbare plants, the transgenic lines had significantly decreased grain size (Table 1), resulting in approximately 7.8% and 11.1% decreases in 1000-grain weight, respectively (Fig. 3d, e). Down-regulation of *GNS4* also led to reduced panicle number in RNAi1 and RNAi2 lines (Table 1).

**Table 1:** Comparison of agronomic traits between NIP and the transgenic lines

	NIP	RNAi1	RNAi2	OE1	OE2
Grain length (mm)	7.51 ± 0.12	6.60 ± 0.35^a^	6.52 ± 0.13^a^	8.01 ± 0.19^a^	7.96 ± 0.17^a^
Grain width (mm)	3.39 ± 0.18	3.15 ± 0.15^b^	3.17 ± 0.22^c^	3.27 ± 0.08^NS^	3.38 ± 0.10^NS^
Grain thickness (mm)	2.41 ± 0.07	2.36 ± 0.06^NS^	2.28 ± 0.06^a^	2.33 ± 0.07^c^	2.39 ± 0.12^NS^
Plant height (cm)	82.74 ± 4.61	72.69 ± 5.39^a^	72.47 ± 5.99^a^	86.50 ± 3.33^a^	91.97 ± 4.01^a^
Panicle length (cm)	21.47 ± 1.38	19.75 ± 1.13^a^	20.41 ± 1.82^c^	23.68 ± 1.87^a^	23.98 ± 1.62^a^
Panicle number per plant	17.00 ± 1.95	14.90 ± 2.08^c^	12.00 ± 2.98^a^	18.09 ± 3.73^NS^	15.73 ± 3.32^NS^
Grain yield per plant (g)	23.63 ± 3.43	10.37 ± 3.82^a^	10.56 ± 3.93^a^	27.60 ± 4.85^c^	27.35 ± 3.86^c^
Biomass yield per plant (g)	51.56 ± 9.57	33.34 ± 8.02^a^	35.13 ± 5.30^a^	64.08 ± 11.03^b^	61.98 ± 10.04^c^

Data are given as means ± s.e. (*n* ≥ 8). Significant at ^a^0.1%, ^b^1% and ^c^5%. NS, not significant. NIP, Nipponbare. RNAi1 and RNAi2 are two independent lines for RNAi analysis, and OE1 and OE2 are two independent lines for overexpression analysis

We also produced overexpression lines of *GNS4* in the Nipponbare background. Two independent positive lines with higher *GNS4* transcript levels (Fig. 3c), OE1 and OE2, showed significantly higher plants, longer panicles, bigger and more grains, compared with those of WT (Fig. 3b, f; Table 1). However, no obvious increase in panicle number was observed in OE1 and OE2 lines (Table 1). At mature stage, we carried out a yield test and found that the elevated expression level of *GNS4* enhanced both biomass yield and grain yield (Table 1).

Together, these results provided persuasive evidences that *GNS4* has large effects on multiple traits, and improves yield production by producing more and bigger grains in rice.

### Mutation of *GNS4* decreased cell size in rice grains

**Fig. 4:**
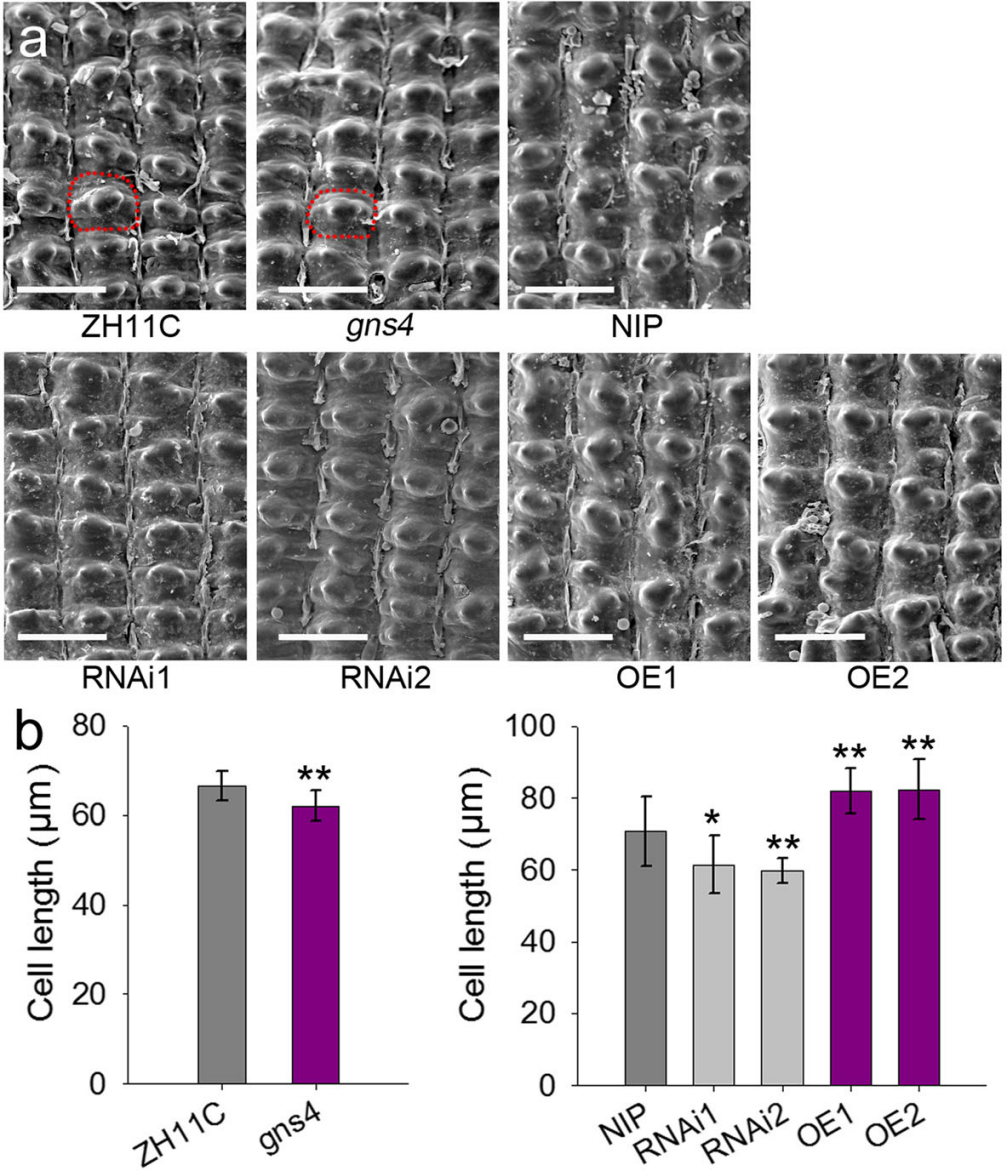
The effect of *GNS4* on cell size of glume. **a** Scanning electron microscope photographs of the glume outer surfaces of mature seeds. The outline of one glume cell in WT and *gns4* hulls, respectively, is boxed by the red dotted line. Bar, 100 μm. **b** Comparison of average length of outer glume surface cells. Data are shown as means ± s.e. (*n* ≥ 10). Significant at **1% and *5%

The BRs are plant steroid hormones that regulate many aspects of plant development, such as organ size and cell elongation (Yang et al. 2011). The *gns4* mutant produced smaller grains than those of WT. The size of the spikelet hull sets an upper limit for sink size and finally determines the grain size (Sakamoto and Matsuoka 2008). To investigate whether cell number or cell size contributes to the difference in grain size, we observed the outer glume epidermal cells under a scanning electron microscope. The outer epidermal cells were smaller in *gns4* than in WT, for example, there was a ~ 6.6% decrease in longitudinal cell length on *gns4* spikelet hulls (Fig. 4). As mentioned above, the grain length was 5.5% shorter in *gns4* than in WT. The SEM analysis of the outer glume epidermal cells of RNAi and overexpression plants showed that the cell length varied with the transcript levels of *GNS4* (Fig. 4). These results indicated that *GNS4* regulates spikelet hull size and grain weights largely by controlling cell elongation.

**Fig. 5:**
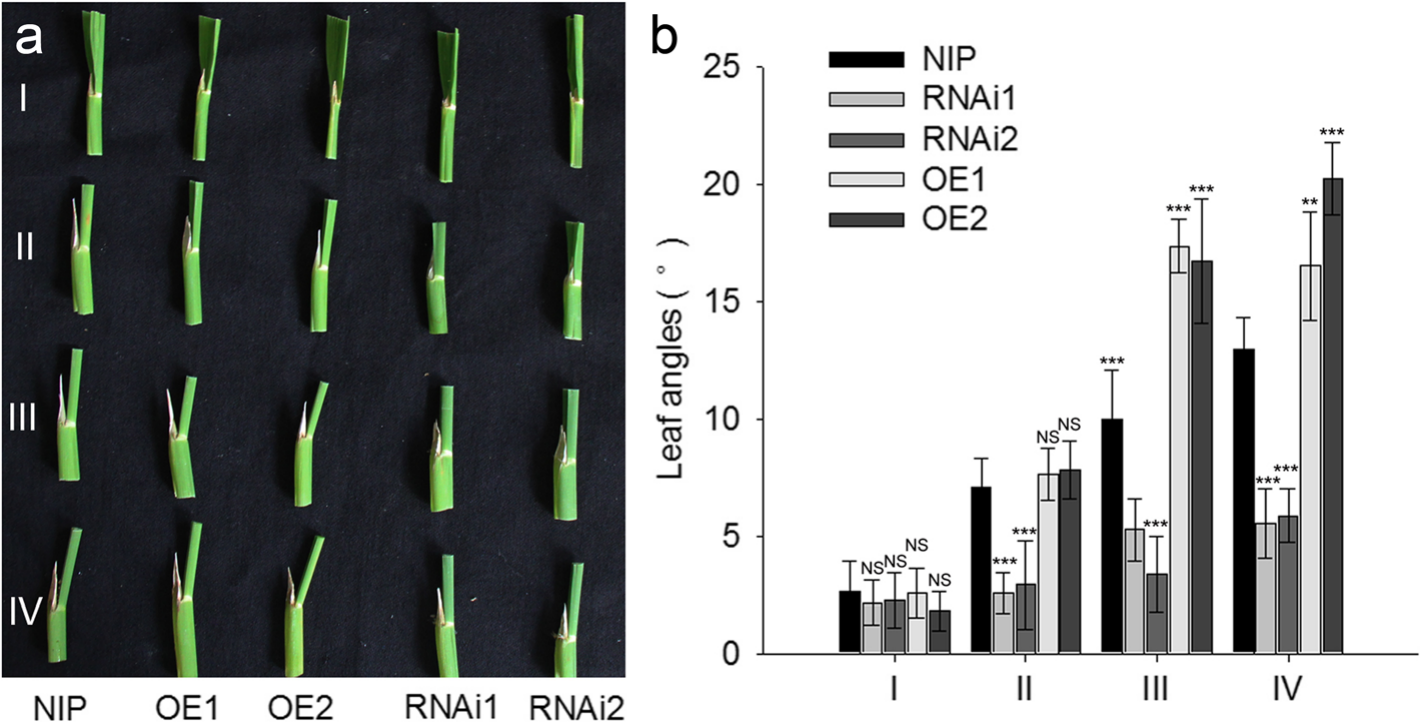
The effect of *GNS4* on leaf angle. **a** Comparison of the lamina joint angle of the flag (I), second (II), third (III), and fourth (IV) leaves (counted from the flag leaf downwards) between Nipponbare and transgenic plants. **b** Quantification of the leaf lamina joint angles. Data are shown as means ± s.e. (*n* = 7). Significant at ***0.1%, and **1%. NS, not significant

Previous studies reported that BR-deficient or BR-insensitive rice mutants exhibit erect leaves (Zhang ea. al., 2014). We examined the lamina joint bending angles and found that down-regulation of *GNS4* led to erect leaves, while *GNS4* overexpression caused an enlarged leaf angle (Fig. 5). We also compared the lamina joint angle of the flag (I), second (II), third (III), and fourth (IV) leaves (counted from the flag leaf downwards) at the heading stage between Nipponbare and transgenic plants. The angles of the bottom leaves were larger than those of the flag leaves (Fig. 5), implying that the effect of *GNS4* on leaf angle increases with leaf age or stage of development.

### Transcript levels of *GNS4*

**Fig. 6:**
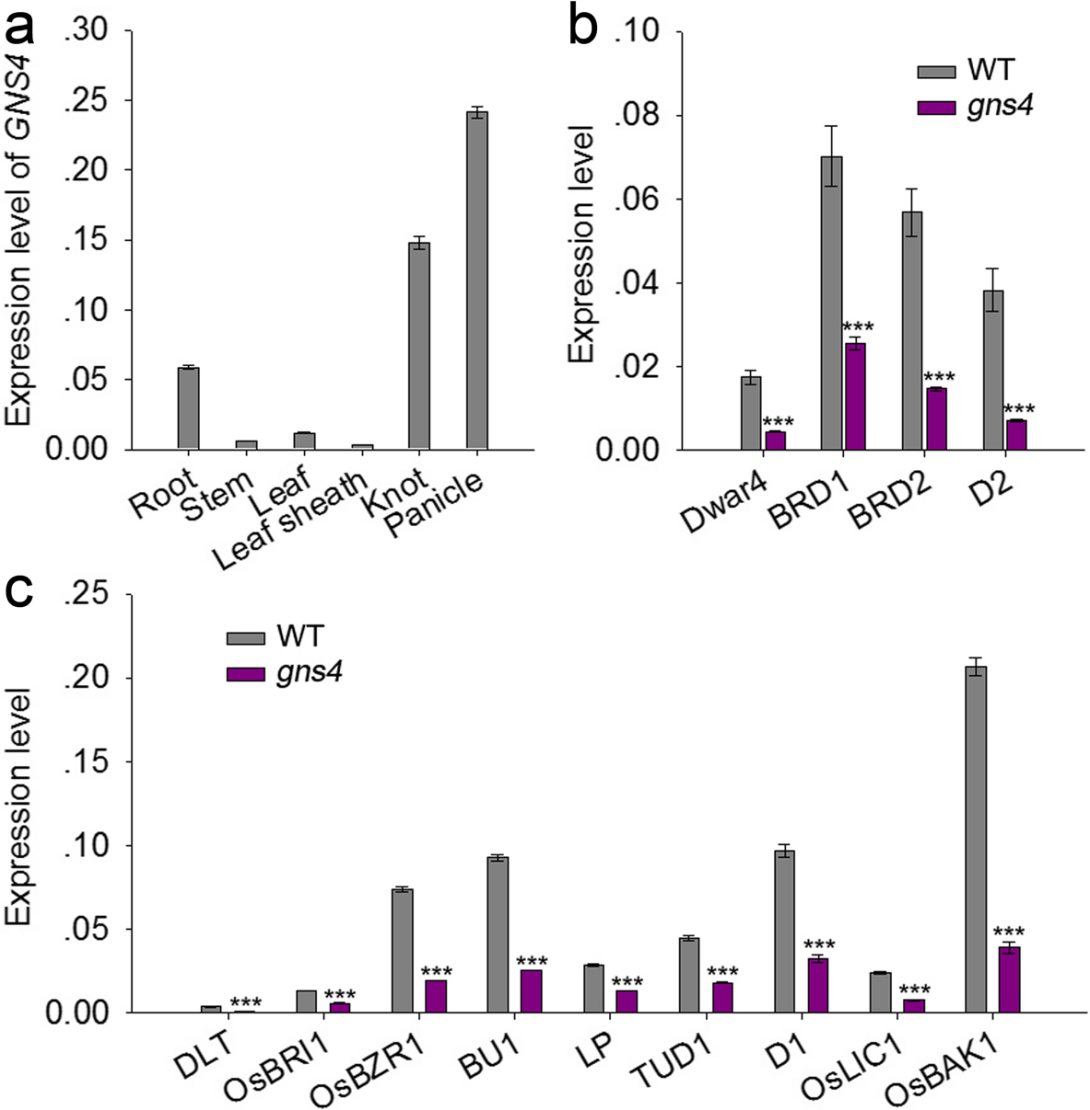
Expression analysis of *GNS4* by qRT-PCR. **a** Relative expression levels of *GNS4* in different tissues. **b** Comparison of transcription level of genes involved in rice BR biosynthesis between WT and *gns4* mutant. **c** Relative transcription levels of the genes related to BR signaling in WT and *gns4* mutant. The expression level of the rice *Actin* gene was amplified as a control. Values are means ± s.e. of four independent experiments

The results of the qRT-PCR analysis indicated that in ZH11C plants, the highest transcript levels of *GNS4* were in the young panicle, with much lower transcript levels in the stem, leaf, and leaf sheath (Fig. 6a). We also examined the transcript levels of four BR biosynthesis genes and nine BR-signaling genes in WT and the *gns4* mutant. Interestingly, no feedback regulation of these BR-related genes was detected. The transcript levels of all 13 detected genes were significantly lower in the *gns4* mutant than in WT (Fig. 6b, c).

### Elevated *GNS4* expression in a high-yielding variety background increases yields

**Fig. 7:**
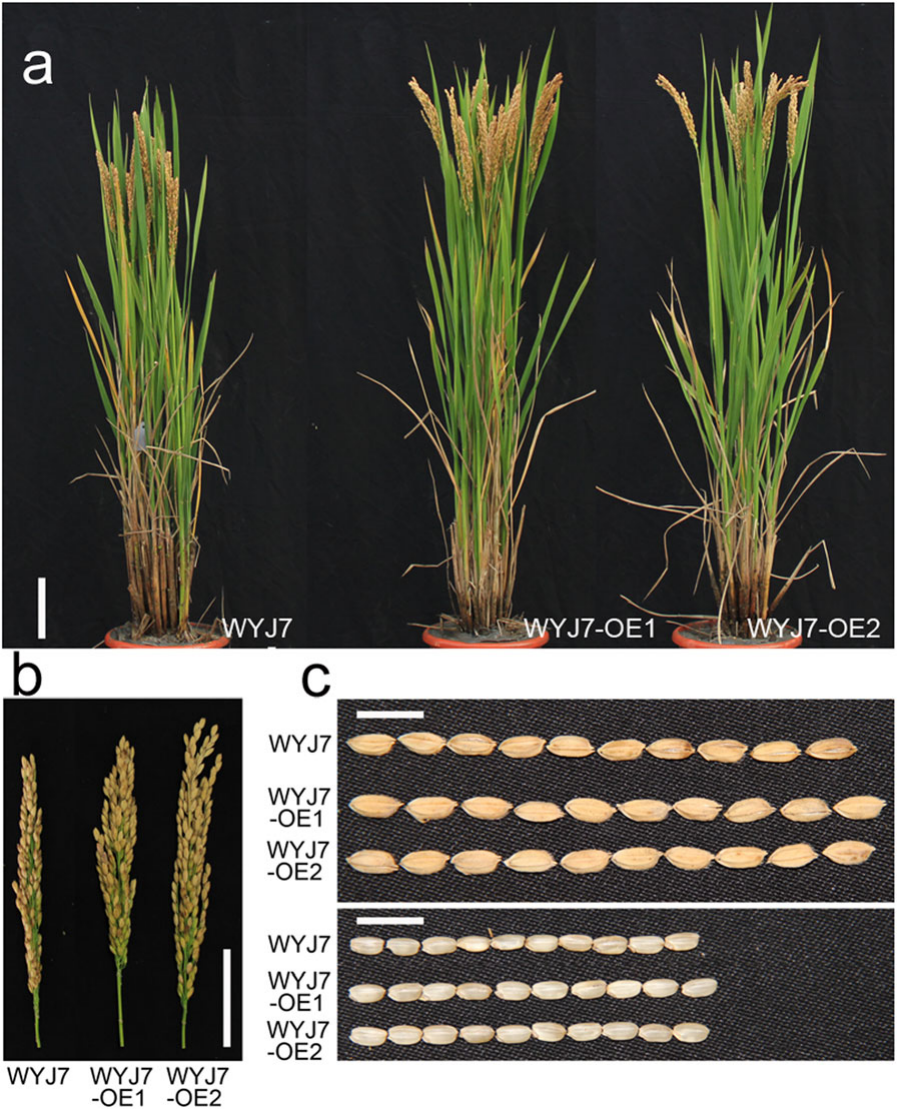
Plant morphology, panicle architecture and grain size of *GNS4*-overexpressing lines in high-yield variety Wuyunjing 7 (WYJ7) background. **a** Mature plant appearance of WYJ7 and the transgenic lines. Bar, 10 cm. **b** Panicle architecture of WYJ7 and the transgenic lines. Bar, 5 cm. **c** Comparisons of grains (top) and brown rice (bottom) between WYJ7 and the transgenic lines. Bars, 1 cm. WYJ-OE1 and WYJ-OE2 are two independent *GNS4*-overexpressing lines

**Fig. 8:**
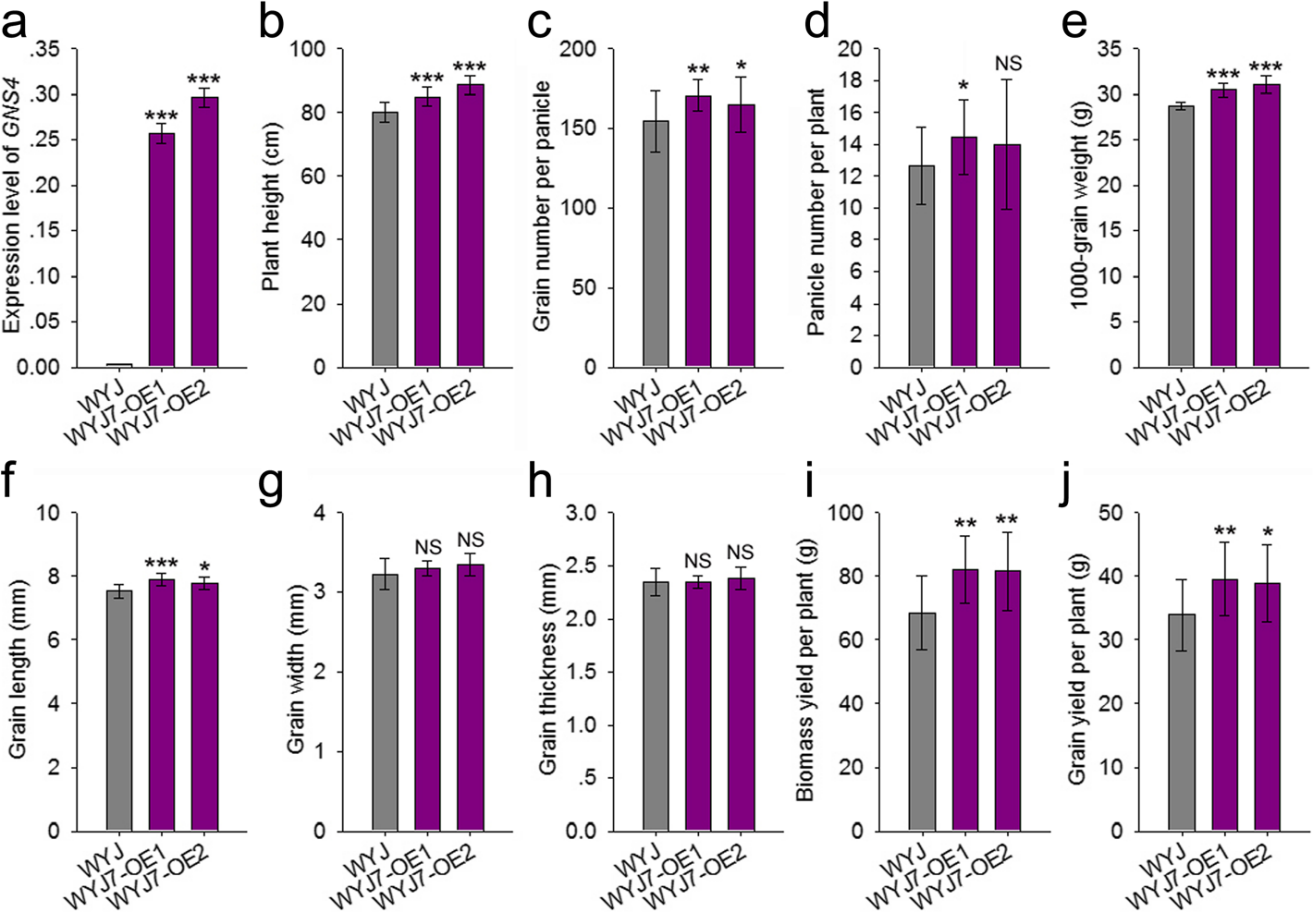
Agronomic traits of *GNS4*-overexpressing lines in high-yield variety Wuyunjing 7 (WYJ7) background. **a** *GNS4* mRNA expression level of WYJ7, WYJ-OE1 and WYJ-OE2 plants. The expression level of the rice *Actin* gene was amplified as a control. Values are means ± s.e. of three independent experiments. **b-j:** Agronomic traits of WT and transgenic lines. **b** Plant height. **c** Grain number per panicle. **d** Panicle number per plant. **e** 1000-grain weight. **f** Grain length. **g** Grain width. **h** Grain thickness. **i** Biomass yield per plant. **j** Grain yield per plant. Traits were measured at the mature stage. Data are given as means ± s.e. (*n* ≥ 10). Significant at ***0.1%, **1% and *5%. NS, not significant. WYJ-OE1 and WYJ-OE2 are two independent *GNS4*-overexpressing lines

As mentioned above, overexpression of *GNS4* in the Nipponbare background led to an obviously enlarged grain size. To further evaluate the potential use of the *GNS4* gene, we generated transgenic lines using Wuyunjing 7 (WYJ7) as the recipient parent. WYJ7 is high-yielding variety that is widely cultivated in Jiangsu Province, China. More than 10 independent transgenic lines were obtained, and two homozygous T_3_ lines with elevated *GNS4* transcript levels were selected for further analysis (Fig. 8a). At maturity, the *GNS4*-overexpressing lines exhibited dramatically improved growth, compared with WYJ7. The plant height of WYJ7-OE1 and WYJ7-OE2 was 6.1% and 10.5% greater, respectively, than that of WYJ7 (Figs. 7a and 8b). The most obvious improvement by *GNS4* overexpression was the enlarged sink size, as reflected by the increased grain number per panicle and larger grains. The grain number per plant in WYJ7-OE1 and WYJ7-OE2 was increased by 10.4% and 6.8%, respectively, compared with that of WYJ7 (Figs. 7b and 8c). The 1000-grain weight of WYJ7-OE1 and WYJ7-OE2 was increased by 5.9% and 7.9%, respectively, compared with that of WYJ7 (Figs. 7c and 8e). And the enlarged grain size mainly resulted from grain length, rather than grain width and thickness (Fig. 8f-h). Compared with WYJ7, the overexpression lines had a little more panicle number per plant (significant in WYJ7-OE1 but not significant in WYJ7-OE2) (Fig. 8d). The biomass yield per plant was remarkably increased in the WYJ7-OE1 and WYJ7-OE2 (Fig. 8i). Finally, the grain yield per plant was improved by 16.5% and 14.6%, respectively, compared with that of WYJ7 (Fig. 8j).

## Discussion

Rice is a very important food crop because it feeds more than half of the world’s population. Grain yield improvement is the main aim for rice breeders. Rice yield potential is determined by biomass and the harvest index. The harvest index of the cultivated rice varieties in China has almost reached its theoretical limit. Improving biomass production is an effective strategy to break through the yield ceiling. Rice biomass depends on plant organ size, which is controlled by genetic factors and environmental conditions. Although numerous genetic determinants of organ growth have been characterized, our understanding of how organ size is regulated is incomplete.

To reveal more genes related to organ size in rice, we characterized a rice mutant, *gns4*, with reduced panicle and grain size. Map-based cloning demonstrated that reduced transcription of *GNS4* caused by an SNP in its promoter region resulted in the mutant phenotype. Down-regulation of *GNS4* by RNAi also resulted in smaller panicles and grains. This finding suggested that *GNS4* plays a positive role in regulating panicle and grain size in rice.

*GNS4* encodes a cytochrome P450 superfamily protein CYP724B1, and is allelic to the previously reported *D11,* which plays roles in BR synthesis (Tanabe et al. 2005). In plants, BRs are essential steroid hormones that regulate diverse processes during plant development, such as stem elongation, vascular differentiation, male fertility, senescence, and responses to various biotic and abiotic stresses (Yang et al. 2011). As described in a previous study, both the *d11–1* and *d11–2* mutants exhibited shortened internodes and produced extremely small round grains. However, the *gns4* mutant did not show this severe phenotype, with only a 5.5% and 9.4% reduction in grain length and grain weight, respectively, compared with those of WT. Molecular detection indicated that an SNP variation in the promoter region slightly decreased the expression level of *GNS4*, leading to the mutant phenotype. These data suggested that *gns4* is a weak mutant of the *GNS4* gene. In rice, a BR-deficient mutant displayed erect leaves, reduced plant height, and decreased tiller number and grain size (Yang et al. 2011). In addition to these common traits, the *gns4* mutant has some distinct traits. For instance, *gns4* has a very short main axis with most of the primary branches clustered at the base of the main axis, and few secondary branches. Together, these results suggested a novel and important role for *GNS4/D11* in regulating inflorescence development.

Because *GNS4* is involved in regulating panicle and grain size, we were interested in whether this gene could be used to improve rice yields. To investigate the function of *GNS4*, we generated several *GNS4-*overexpressing lines in Nipponbare background. Strong expression of *GNS4* in the transgenic lines was detected by qRT-PCR (Fig. 3c). At the heading and mature stages, rice plants harboring the overexpression construct outgrew Nipponbare plants (Fig. 3), indicating that *GNS4* controls vegetative growth. When grown in paddies, the biomass of *GNS4-*overexpressing lines was greater than that of Nipponbare (Fig. 3; Table 1). Multiple sink-related traits, including grain size and grain number per panicle, were enhanced in the *GNS4-*overexpressing lines. As anticipated, the grain yield per plant was increased by 16.8% and 15.7%, compared with that of Nipponbare. Next, we created *GNS4-*overexpressing lines using WYJ7, a high-yielding variety, as the recipient. These *GNS4-*overexpressing lines also showed significant biomass and grain yield improvements (Fig. 8i, j). Taken together, these results indicated that *GNS4* has multiple beneficial effects on grain yield components, and is valuable for high-yield rice breeding.

Recently, Wu et al. (2016) found that the overexpression line (OE-*CPB1*) of *CPB1/D11* under the control of the maize ubiquitin promoter showed increased grain length and 1000-grain weight. However, there was no significant increase in the grain yield per plant of OE-*CPB1* plants as compared with WT, because the transgenic plants showed profound changes in plant architecture, such as larger leaf angles and narrower leaves. Transgenic plants expressing *CPB1/D11* under the control of panicle-specific promoters from *DEP1* and *TH1* produced larger seeds and increased grain yields without changes in other agronomic traits, such as grain number per panicle. In this study, the *D11-*overexpressing lines in both the Nipponbare and WYJ7 backgrounds produced larger seeds, more grains, and increased yields, compared with those of WT. We noticed that the *CPB1/D11* transcripts in OE-*CPB1* lines accumulated more than 450-fold than in WT. In this study, however, there is only 30.0- to 93.6-fold increase in *GNS4/D11* expression in the overexpression transgenic lines (Figs. 3c and 8a). As a result, the phenotypic variations in our data are not as big as Wu’ results. We speculate that the yield-increasing effect of *D11* may be determined by its expression pattern, and may also be affected to some extent by genetic backgrounds.

## Conclusions

The rice *gns4* mutant showed reduced organ size, fewer grains per panicle, and smaller grain. Map-based cloning investigated that the *GNS4* gene, encoding a cytochrome P450 protein, is allelic to *DWARF11* (*D11*). Morphological and cellular analyses suggested that *GNS4* positively regulates grain size by promoting cell elongation. Elevated expression level of *GNS4* significantly increased 1000-grain weight and grain number per panicle, and subsequently enhanced grain yields in both the Nipponbare and Wuyunjing7 (a high-yielding cultivar) backgrounds. These results suggest that *GNS4* is key target site to increase yields in rice breeding programs.

## Methods

### Plant materials

The spontaneous mutant, *gns4*, was identified from *japonica* rice Zhonghua 11C (ZH11C). The mutant was self-pollinated for several generations until the mutation was genetically proven to be truly inherited.

### Genetic analysis and fine mapping of *GNS4*

For genetic analyses, an F_2_ population derived from a cross between *gns4* and 9311, an *indica* variety, was grown in paddy fields under natural conditions. This segregating population was used for fine mapping of the *GNS4* locus. Recessive individuals in the F_2_ segregating population were used to screen recombinants. To fine-map *GNS4*, several polymorphic InDel molecular markers were developed based on sequence differences between the *indica* variety 9311 and the *japonica* variety Nipponbare (Additional file 1: Table S1), according to data published at the NCBI (http://www.ncbi.nlm.nih.gov). Candidate genes were amplified and sequenced using gene-specific primers.

### RNA extraction, complementary DNA synthesis, and qRT-PCR

Total RNA was isolated from various organs using an RNA extraction kit following the manufacturer’s instructions (Beijing Tiangen Biotechnology Co. Ltd., Beijing, China; www.tiangen.com/). First-strand cDNA was reverse-transcribed from approximately 1 μg gDNase-treated RNA using a FastQuant RT Kit following the manufacturer’s instructions (Beijing Tiangen Biotechnology Co. Ltd.). Gene transcript levels were determined by quantitative reverse transcription-PCR (qRT-PCR) with the rice *Actin* gene as the control. Each qRT-PCR was performed in a total volume of 25 μL containing 2 μL cDNA, 0.2 mM each primer, and 12.5 μL 2 × SYBR green PCR master mix (Takara, Dalian, China; http://www.takara.com.cn). The qRT-PCR was carried out using an ABI ViiA7 real-time PCR system using the following program: 95 °C for 3 min, then 40 cycles of 94 °C for 30 s, 55 °C for 30 s, and 72 °C for 40 s. Relative gene transcript levels were calculated using the 2^-ΔΔ^C_T_ method.

### Vector construction and plant transformation

For the complementation test, the promoter (a DNA fragment ~2 kb upstream of translation start site) of *GNS4* was amplified from ZH11C genomic DNA. The full coding region of *GNS4* was also cloned. Both segments were then cloned into the binary pCAMBIA1301 vector to generate a construct in which *GNS4* was driven by its native promoter. The full coding region of *GNS4* was amplified from ZH11C cDNA and then inserted into the p1301UbiNOS vector to generate an overexpression construct in which *GNS4* gene was controlled by a constitutively expressed maize ubiquitin promoter (Zhou et al. 2009). This overexpression construct was transformed into *japonica* varieties Nipponbare and Wuyunjing 7. For RNAi analysis, a DNA fragment of LOC_Os04g39430 was amplified and then cloned into the pMD18-T vector (Takara), before being cloned into the BamH I/Spe I and Bgl II/Xba I sites of the p1022 vector. Then, the stem-loop fragment was cloned into the p1301UbiNOS vector (Zhou et al. 2009). All the constructs were transformed into the recipient lines by *Agrobacterium tumefaciens* (strain EHA105) mediated transformation.

### Evaluation of agronomic traits

Forty plants of each line were grown in the experimental field of Yangzhou University (E119°25′/N32°23′), from May through October in 2016. The distance between the plants in a row was 17.0 cm, and the distance between rows was 23.3 cm. Nitrogen (225 kg ha^−1^ as urea), together with phosphorus (50 kg ha^−1^ as single superphosphate) and potassium (60 kg ha^−1^ as KCl), were applied after transplanting. Field management and disease and pest control followed the standard procedures to prevent yield loss during the growth period.

All traits were evaluated at the mature stage. Plant height was measured from the ground surface to the tip of the tallest panicle. Panicle number per plant was the number of effective panicles with 10 or more grains. We also counted the grain number per panicle, and measured 1000-grain weight, grain yield per plant, and biomass per plant. Paddy grains were dried naturally after harvesting and stored at 37 °C for at least 1 week before testing. Fully filled grains were used for measurements. The independent sample *t-test* program of SPSS 10.0 for Windows was used to compare mean values among the mutants, overexpression and RNAi lines, and WT.

### Morphological and cellular analyses

The glume outer surfaces of mature seeds were directly observed under a scanning electron microscope (SEM). The length of cells on the spikelet hull was measured using Image J software.

## Additional file

Additional file 1: Table S1. Primers used in this study. (PDF 37 kb)
