# Supplementary material for: GNS4, a novel allele of DWARF11, regulates grain number and grain size in a high-yield rice variety
Source: Rice (N Y). 2017 Jul 20;10:34. doi: 10.1186/s12284-017-0171-4 (PMC5519514; doi:10.1186/s12284-017-0171-4)
Supplement: Additional file 1: Table S1. — Primers used in this study. (PDF 37 kb) [file 12284_2017_171_MOESM1_ESM.pdf]

Supplemental Table S1. Primers used in this study.

Primers	Forward (5'-3')	Reverse (5'-3')	Purpose
LYH-52	TCTTCTCACTATTCGCTTCG	TCGTCATCGCTTGAGGTT	Mapping
LYH-54	GTTGTACGGCACGCTGAT	TTTATTTACAGGGTGAGATGG	Mapping
LYH-71	TTTGGCTAGGTTTGACGG	AATGGGCCTTTGATTGG	Mapping
LYH-73	TTTGGCTAGGTTTGACGG	AATGGGCCTTTGATTGG	Mapping
LYH-84	GAAGAAGTAGATAGGAGGAGGA	AGATCAAGATCAGCCCAAG	Mapping
LYH-91	CGAAATCACATCTCAATCC	TCTTGCGTTTCTGGCTAT	Mapping
39430HB-Pro	aaaGAATTCTGGTGGCCATTGTAGGAGTAGGTCT	aaaGGTACCCTTACCTATAGCCTCACACCAGG	Complementation test
39430HB-CDS	aaaGGTACCATGGTGGGAGGAGAGCTTGTGCT	aaaGGATCCTCAGGATTCAGAGCAAAGTGGCTCG	Complementation test
RNAi1	aaaGGATCCGGTGAGACGCTGAGGTT	aaaACTAGTCCTGAGCCTCTTGTGGT	RNAi analysis
OX1	aaaGGATCCATGGTGGGAGGAGAGCTTGTGCT	aaaGGTACCTCAGGATTCAGAGCAAAGTGGCTCG	Overexpression analysis
Real_GNS4	TTGCTTTGCTGCTGACC	TCCTGAGCCTCTTGTGGT	qRT-PCR
Real_Actin	GATGACCCAGATCATGTTTG	GGGCGATGTAGGAAAGC	qRT-PCR
